# Lectin Engineering, a Molecular Evolutionary Approach to Expanding the Lectin Utilities

**DOI:** 10.3390/molecules20057637

**Published:** 2015-04-27

**Authors:** Dan Hu, Hiroaki Tateno, Jun Hirabayashi

**Affiliations:** 1 Institute of Traditional Chinese Medicine and Natural Products, College of Pharmacy, Jinan University, Guangzhou 510632, China; E-Mail: thudan@jnu.edu.cn; 2 Biotechnology Research Institute for Drug Discovery, National Institute of Advanced Industrial Science and Technology, Central-2, 1-1-1, Umezono, Tsukuba, Ibaraki 305-8568, Japan; E-Mail: h-tateno@aist.go.jp

**Keywords:** lectin engineering, molecular evolution, carbohydrate specificity, scaffold, error-prone polymerase-chain reaction (PCR)

## Abstract

In the post genomic era, glycomics—the systematic study of all glycan structures of a given cell or organism—has emerged as an indispensable technology in various fields of biology and medicine. Lectins are regarded as “decipherers of glycans”, being useful reagents for their structural analysis, and have been widely used in glycomic studies. However, the inconsistent activity and availability associated with the plant-derived lectins that comprise most of the commercially available lectins, and the limit in the range of glycan structures covered, have necessitated the development of innovative tools via engineering of lectins on existing scaffolds. This review will summarize the current state of the art of lectin engineering and highlight recent technological advances in this field. The key issues associated with the strategy of lectin engineering including selection of template lectin, construction of a mutagenesis library, and high-throughput screening methods are discussed.

## 1. Introduction

Carbohydrates are intricate information-carrying biopolymers that have attracted increasing interest in the post-genomic era. Dramatic changes in glycosylation features have been observed in various key biological events such as embryogenesis, differentiation, and tumorigenesis [1,2]. The systematic analysis of all glycan structures of a given cell or organism, *i.e.*, glycomics, is therefore critical to understanding the roles of carbohydrates and helpful in the identification of potential biomarkers [3,4].

Various methods have been developed for structural characterization of glycans [5]. Among them, mass spectrometry (MS) is the most widely used technique, which is not only able to give relatively detailed structural information of glycans, but is also useful for the simultaneous analysis of multiple glycans of complex samples, enabling their high-throughput analysis. In most cases, however, this method requires prior liberation of glycans from glycoconjugates (e.g., glycoproteins) and their separation by high-pressure liquid chromatography (HPLC) or capillary electrophoresis [6], which significantly decreases the speed of the analysis. In addition, the difficulty in interpretation and assignment of complex MS spectra due to the presence of diverse glycan isomers is still a major bottleneck of this technique. Complementary approaches to glycomics are thus needed, which are distinct from the MS-based principle.

HPLC is a conventional method for glycome profiling based on the separation of labeled oligosaccharides. Briefly, glycans from complex biological samples are released by either PNGase F treatment (for *N*-linked glycans) or β-elimination (for *O*-linked glycans), and tagged at the reducing end with a fluorescent label such as 2-aminobenzamide, 2-aminopyridine and anthranilic acid. The labeled glycans are then purified and analyzed using HPLC in various modes to identify their structures with reference to authentic standards. The resulting HPLC traces can also be used to compare the glycomic profiles of multiple samples. A recent study utilized this procedure to compare quantitatively the glycomes of undifferentiated and differentiated cells, and demonstrated that the linkage of sialic acid on *N*-linked glycans was dramatically changed from α2-3 to α2-6 upon induction of pluripotency [7]. However, because of the diversity and complexity of glycan structures, even a single peak in an HPLC profile often contains multiple glycans, which will complicate the data interpretation. Further analysis in combination with exoglycosidase digestion is often used to obtain specific structural information from this technique [5,7]. To assist the interpretation and assignment of HPLC-glycan profiles, a database (GlycoBase) and an automatic analytic tool (autoGU) were recently developed, which greatly facilitate the glycan analysis by HPLC [8].

Lectins, defined as sugar-binding proteins, are useful reagents for structural characterization of glycans. Until now, lectins have been extensively used in cell typing, histochemical staining, and glycoprotein fractionation [4]. Recently, a novel technique called lectin microarray, in which a panel of well-defined lectins is immobilized onto a solid support, has been successfully used for high-throughput analysis of complex carbohydrates included in serum glycoproteins and whole cells [9,10,11]. Despite these successes, the relatively low affinity of lectins for glycans has significantly reduced the detection sensitivity when used as analytical reagents. To overcome this problem, Stevens *et al.* made use of multivalent interactions between lectins and glycans frequently found in biological systems, and successfully determined the specificity of influenza hemagglutinin (HA) to its sialic acid ligand in a glycan microarray assay through clustering of recombinant HA [12]. Similarly, Kawasaki *et al.* prepared a tetrameric lectin by mixing biotinylated lectin with streptavidin to enhance the weak sugar-binding activity. This approach was successfully applied to the analysis of sugar-binding specificities of several endoplasmic reticulum-resident lectins, of which affinities are generally weak [13,14,15,16,17].

In addition to adopting a multimerization strategy to increase the detection sensitivity, Kuno *et al* recently developed a highly sensitive system based on the evanescent-field activated fluorescence detection principle, which allows quantitative detection of even weak lectin-carbohydrate interactions (*K_d_* > 10^−6^ M) under equilibrium conditions without any washing process [9]. Using this approach, Tateno *et al.* demonstrated that a recombinant lectin probe, rBC2LCN, specifically bound to undifferentiated pluripotent stem cells, but not differentiated somatic cells; this has now been developed as a useful undifferentiation marker [2,18,19]. To obtain information about which proteins glycans of interest are attached to, Kuno *et al.* established an approach called antibody overlay-lectin microarray, which has been applied to the investigation of a useful hepatic fibrosis marker, α1-acid glycoprotein probed with *Maackia amurensis* lectin and *Aspergillus oryzae* lectin [20].

Despite the achievements described above, several issues must be addressed to make lectins more useful tools for glycomics [21]. First, most of the lectins used previously are of plant origin, having some inherent problems, such as inconsistent activity and unreliable availability. Second, a currently available lectin set has an apparent drawback in its “repertoire”, lacking some critical probes for less common glycan structures, such as those for sulfated glycans. Third, lectins often have broad specificity, which complicates the interpretation of data using complex samples. Engineering of lectins based on existing scaffolds for improved properties including specificity, affinity and stability would be of great practical value to solve the above problems [22].

## 2. Current State of the Art of Lectin Engineering

**Scheme 1:**
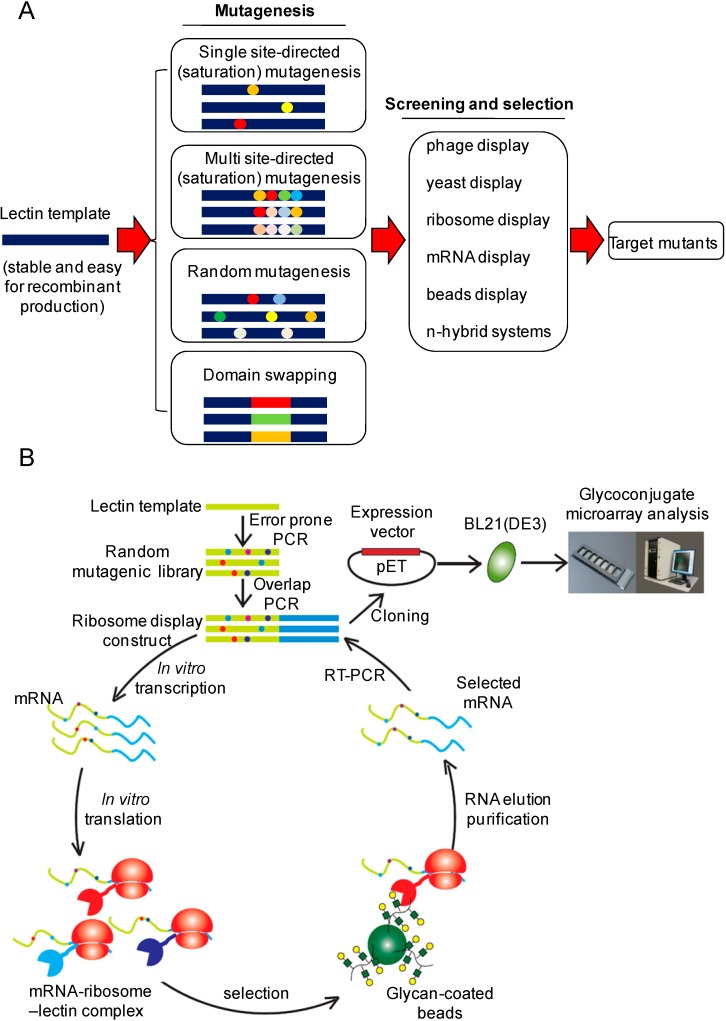
(**A**) A current state of the major procedures of lectin engineering, comprising construction of mutagenic lectin libraries and a subsequent process for screening/selection of target mutants. (**B**) A strategy developed in our laboratory for lectin engineering, which is based on error-prone PCR for library construction and a reinforced ribosome display method for selection of target mutants.

Lectin engineering is a kind of protein engineering technology. Many methods including site-directed mutagenesis, site-directed saturation mutagenesis, random mutagenesis, DNA (exon) shuffling are thus directly applied to lectin engineering (Scheme 1). However, due to the relatively weak lectin–glycan interaction, modifications of these methods are sometimes necessary to fulfill the requirements for lectin engineering.

### 2.1. Engineering of L-Type Lectins

L-type lectins were first found in the seeds of leguminous plants, and are now used in a wide range of biomedical and analytical procedures. Though these lectins exhibit strong homology with respect to their primary structures, called a “jelly-roll fold”, they show significant difference in their sugar-binding specificities, and are categorized based on monosaccharide specificity; e.g., Gal-specific, Man/Glc-specific and L-Fuc-specific lectins [23]. Such promiscuous properties of L-type lectins have made them good scaffolds for engineering novel lectins with target specificity.

Structure-based site-directed mutagenesis is the most widely used method for lectin engineering. Guided by the crystal structure of *Erythrina corallodendron* lectin (EcorL), which preferentially binds to Gal derivatives with bulky substituents at C-2 such as *N*-dansylgalactosamine, Arango *et al.* conducted site-directed mutagenesis and obtained a mutant specific for Gal [24]. Jordan *et al.* later used the same strategy for engineering of lima bean lectin, an A-trisaccharide-specific lectin, and generated two mutants—C127Y and H128P—both of which exhibit altered specificity for A disaccharide (GalNAcα1-3Gal) and Forssman disaccharide (GalNAcα1-3GalNAc), respectively [25].

Thamotharan *et al.* also performed site-directed mutagenesis of EcorL and prepared a mutant with enhanced affinity for GalNAc by a single amino acid substitution of Tyr106 to glycine [26]. Peanut agglutinin is a clinically important lectin due to its high affinity for the tumor associated Thomsen-Friedenreich antigen (T-antigen). To engineer a practical probe with improved specificity for T-antigen, Adhikari *et al.* focused on Asn41 of this lectin and tailored a mutant N41Q with a four-fold increase in the binding affinity for T-antigen [27].

In addition to the point mutagenesis described above, a strategy by region replacement was also adopted. Yamamoto *et al.* substituted the metal-binding region of the Gal-specific lectin from *Bauhinia purpurea* (BPA) with the corresponding region of the Man-specific lectin from *Lens culinaris* (LCA). The resulting chimeric lectin acquired affinity for Man-BSA and high Man-type glycopeptides, while it lost its original affinity for Gal [28,29]. Nishiguchi *et al.* used a similar strategy to replace the metal-binding site of Gal-binding lectin from the bark of *Robinia pseudoacacia* (RBL) with the corresponding region of LCA. In this case, the RBL mutant agglutinated rabbit erythrocytes. However, this hemagglutination was not inhibited by Man, but rather by Gal [30]. This discrepancy suggested that additional regions other than the nonapeptide in RBL contribute to the sugar-binding specificity.

Phage display is an *in vitro* high-throughput screening technique widely used in protein engineering. Yamamoto *et al.* used this method in the screening of a lectin library constructed by introducing random mutations in the carbohydrate-binding loop of BPA [31]. Several phage clones with an affinity for Man or GlcNAc were isolated. Later, Maenuma *et al.* adopted a similar strategy to randomize six amino acids in loop C of *Maackia amurensis* hemagglutinin (MAH) and isolated 10 novel mutants with different affinity for glycophorin A and monosialyl-T antigen [32]. Based on the fact that all the mutations found in these MAH mutants occurred at two positions, *i.e.*, Gly131 and Ser133, they further conducted a saturation mutagenesis focused on Gly131 and Ser133, and obtained a further 35 MAH mutants. This set of MAH mutants has been shown to be useful for profiling various cells based on the variations of the surface glycans [33].

Though L-type lectins have been extensively studied in the past, the successfully tailored probes are relatively limited from a practical viewpoint, because L-type lectins often undergo post-translational processing in plants, which the *Escherichia coli* system lacks. Therefore, they have substantial difficulty in their expression in *E. coli*, often forming insoluble aggregates [34]. Thus, engineering of lectins other than L-type lectin of scaffolds should be considered if the use of bacterial expression systems is mandatory.

### 2.2. Engineering of Galectins

Galectins are a family of lectins that are defined by an evolutionarily conserved β-galactoside-binding site [35,36]. The carbohydrate-recognition domain (CRD) of galectins consists of ~130 amino acids, which fold into a β-sandwich structure comprising two anti-parallel β-sheets (designated F and S-sheets), with the concave surface of the S-sheet forming a groove to accommodate the glycan ligands. To date, 10 human galectins have been discovered and are considered to play important roles in various physiological processes, such as development and immunology. This makes them promising therapeutic reagents for the treatment of some human disorders [37]. Thus, initial engineering of galectins has been focused on improving their productivity and stability for pharmaceutical potential.

Galectin-9 is a tandem-repeat-type molecule consisting of two CRDs and serves as a novel type of immune modulator; however, it is sensitive to proteolysis due to the presence of a relatively long and flexible linker peptide between the two CRDs, which has greatly hindered its clinical use. Nishi *et al.* prepared mutant proteins by serial truncation of the linker peptide and obtained a mutant designated G9Null lacking the entire linker peptide, which is highly stable against proteolysis while retaining its biological activities [38]. Later, Itoh *et al.* conducted further engineering of G9Null to increase its solubility, and tailored a mutant exhibiting a 400% increase in solubility and yield without an adverse effect on its biological activity by deleting 10 amino acids as well as substituting a single amino acid of the remaining linker peptide [39]. Wang *et al.* focused on the two cysteine residues (Cys57 and Cys75) in galectin-2, and found that substitution of Cys57 with methionine significantly increased the soluble production of this lectin in *E.* *coli* without affecting its biological activity, and also facilitated the site-directed PEGylation at the remaining Cys75 [40]. A further study conducted by Kopitz *et al.* [41] demonstrated that PEGylation of galectins could markedly reduce their sugar-binding properties, which should be kept in mind when engineering galectins by site-directed mutation and PEGylation.

**Figure 1:**
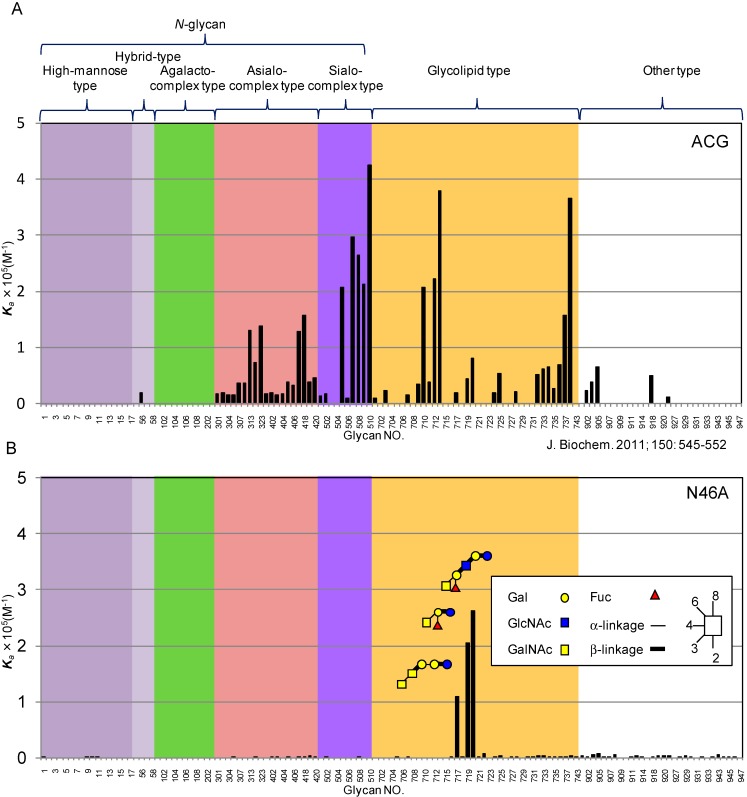
Quantitative analysis of the affinity of N46A to 129 kinds of pyridylaminated (PA) oligosaccharides by frontal affinity chromatography. (**A**) Binding profile obtained for wild-type ACG (data originally from Imamura *et al.* [43]). (**B**) Binding profile of N46A mutant (data originally from Hu *et al.* [45]). For the list of PA-oligosaccharides, see Hu *et al.* [45].

In addition to pharmaceutical applications, some fungal galectins are good targets of engineering for glycan structural characterization. Galectin isolated from *Agrocybe cylindricea* (ACG) has been shown to recognize a variety of glycan epitopes, which include not only typical β-galactosides, such as Galβ1-3/4GlcNAc (LacNAc) and Galβ1-3GalNAc (T antigen), but also many other derivatives, in which the C-3 position of the terminal β-Gal is substituted with Siaα2-3, Sulfo-3, or GalNAcα1-3 [42,43,44]. Recombinant ACG is stable and can be easily produced in *E. coli*, allowing it to be a good scaffold for lectin engineering. In fact, Imamura *et al.* recently conducted saturation mutagenesis in an effort to tailor a Siaα2-3-specific lectin from this galectin [43]. To reduce the background affinity to LacNAc, they focused on Glu86, a residue involved in both terminal Gal and penultimate GlcNAc-binding in the context of LacNAc. Though they succeeded in impairing the background binding to β-Gal, the overall affinity for Siaα2-3-linked glycans, especially those with a single α2-3-linked Sia residue, was also reduced significantly. Recently, Hu *et al.* targeted all of the β-Gal-binding residues of ACG by substituting each with alanine (alanine mutagenesis), and analyzed their binding activity by a high-throughput screening method using an advanced technology, evanescent-type glycoconjugate microarray [45]. As a result, a single amino-acid substitution at position 46 (N46A) in ACG was found to show a dramatic change in the specificity profile: it acquired enhanced activity for GalNAcα1-3Galβ-containing glycans, while totally eliminating the original affinity to β-galactosides. This change was attributed to the disruption of the *cis*-*trans* isomerization involving Pro45 located on an extra loop unique to this galectin [46] (Figure 1). In addition, a single amino-acid substitution of Glu at position 86 with Ala (E86A) in ACG conferred a highly specific binding for 3'-sulfo-Galβ1-4GlcNAc (3'S-LacNAc). The derived E86A mutant could behave as a useful probe for the detection of 3'S-LacNAc-containing glycans, which are relatively rare in nature, and thus, only poor information of this glyco-epitope has been available to date [47].

Yeast surface display (YSD) is also a high-throughput approach widely used in protein engineering for improved secretion, stability and affinity, but has never been applied to lectin engineering. Ryckaert *et al.* used YSD to present galectins on the yeast surface in a multivalent format, and demonstrated that YSD could be used for efficient fishing of target mutants from a complex library [48]. This technology seems to be especially useful for engineering lectins not suitable for *E. coli* expression.

### 2.3. Engineering of R-Type Lectins

R-type lectins containing a ricin-B chain-like carbohydrate recognition domain (R-type domain) are widely distributed in bacteria, plants, and animals. The R-type domain has a three-lobed organization and folds into a β-trefoil structure consisting of α, β, and γ sub-domains. Each sub-domain can be an independent binding site, but in most R-type lectins only one or two of these sub-domains retain the conserved amino acids required for sugar-binding [49]. Notably, most of the R-type lectins bind Gal/GalNAc, but some have evolved to bind their derivatives; e.g., Neu5Acα2-6Gal/GalNAc [50,51], 6S-Gal [52] and 4S-GalNAc [53]. The fact that R-type lectins are capable of recognizing a diversity of glycan epitopes with a similar structure suggests that it could be a good scaffold for engineering probes with altered activity.

**Figure 2:**
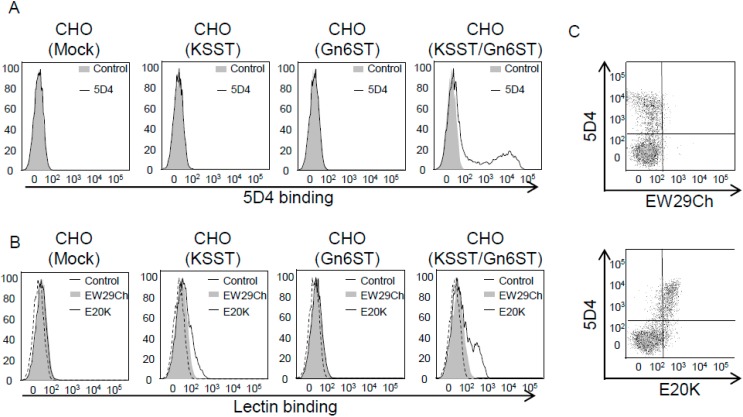
Binding of keratan sulfate-specific antibody 5D4, EW29Ch and its mutant E20K to Chinese hamster ovary (CHO) cells, in which 6S-Gal-catalyzing enzymes were over-expressed. The CHO cells were transfected with keratan sulfate 6-*O*-sulfotransferase (KSST) or GlcNAc 6-*O*-sulfotransferase (Gn6ST) or co-transfected with KSST and Gn6ST for 24 h. The binding of 5D4 (**A**), wild-type EW29Ch, and E20K (**B**) to these transfected cells was investigated by flow cytometry. (**C**) shows the double staining of KSST and Gn6ST co-transfected cells with 5D4 and EW29Ch or E20K. E20K mutant shows enhanced binding to CHO cells, to which KSST and Gn6ST were introduced so that the cells express 6'S-LacNAc structure on the cell surface. Original data are from Hu *et al.* [22].

EW29Ch is the C-terminal domain of earthworm 29-kDa Gal-binding protein (EW29), which contains two R-type domains in tandem [54]. Recombinant EW29Ch is stable and can be easily produced in *E. coli* in a large amount, allowing it to be used for the purpose of engineering. Yabe *et al.* recently adopted a 'natural evolution-mimicry' strategy to tailor a novel α2-6-linked Sia-binding lectin, designated SRC (meaning Sia-specific R-type lectin EW29Ch), from Gal-specific EW29Ch by a procedure consisting of error-prone PCR and a reinforced ribosome display method [55]. To further enhance the affinity of SRC, a tandem repeat of SRC, called SRC2, was engineered, which exhibited potent affinity for α2-6-sialylated *N*-glycans (*K*_d_, ~10^−6^ M) [56]. Despite these successes, the general efficiency of this system for selecting target mutants is low. Hu *et al.* recently refined the strategy by substantially reducing the background noise (*i.e.*, false positive clones) with the inclusion of competent oligosaccharides during the selection procedure [22]. In addition, incorporation of the above-mentioned high-throughput glycoconjugate microarray greatly empowered the strategy in terms of throughput (for details, see Section 3.2. Methods for Mutagenesis). In fact, after only two rounds of selection processes targeting 6'-sulfo-Galβ1-4GlcNAc (6'S-LacNAc), 20 mutants gave positive signals on the screening, and 12 out of them were successfully expressed in *E. coli*. Finally, eight clones proved to show significant affinity for 6'S-LacNAc, which the parental EW29Ch lacked. Thus, we successfully engineered a galactose (Gal)-specific lectin EW29Ch to a mutant (E20K) capable of binding to 6S-Gal (Figure 2).

R-type lectins form a large and extensive family. In fact, some have a long history as useful tools, such as *Sambucus sieboldiana* agglutinin, *S. nigra* agglutinin, and *Viscum album* agglutinin. This implies that the R-type lectin scaffold is promising for engineering practical tools for future glycan analysis.

### 2.4. Engineering of C-Type Lectins

C-type lectins are Ca^2+^-dependent sugar-binding proteins containing a C-type lectin domain (CTLD) consisting of 110–130 amino acid residues. CTLD comprises a double-looped, two-stranded antiparallel β-sheet formed by the N- and C-terminal residues connected by two α-helices and a three-stranded antiparallel β-sheet. The C-type lectin fold is a rigid scaffold that can accommodate a variety of glycan structures. This fact implies that CTLD serves as a versatile scaffold for lectin engineering.

Based on the difference in key conserved residues between Man-binding and Gal-binding CTLDs, Drickamer *et al.* replaced the sequence shared by Man-binding proteins with the corresponding regions found in Gal-binding proteins. The resulting mutant has shifted its basic specificity from Man to Gal [57] as has been seen for engineering of L-type lectins described above (Section 2.1). Man-binding lectin (MBL) is an endogenous C-type lectin circulating in a first line host defense against a wide variety of pathogens, such as viruses. Therefore, MBL can be a potential reagent for viral infection therapy. In this regard, Michelow *et al.* and Chang *et al.* developed a less complex chimeric fusion protein containing the CRD of MBL and the collagenous domain of L-ficolin (L-FCN), which showed similar ligand recognition and enhanced effector functions through the collagenous domain [58,59]. However, MBL itself has a relatively complex quaternary structure, which makes this lectin disadvantageous for large scale production.

C-type lectins are widely expressed in various immune cells and are shown to play important roles in immune response to pathogens. The engineering of C-type lectins could thus be an important way to create compounds for disease treatment.

### 2.5. Engineering of β-Propeller Lectins

β-propeller lectins are a set of sugar-binding proteins consisting of repetitive characteristic structural units called “blades” that are tightly packed into a circular way to form an intact “propeller”. Though all blades have similar structures consisting of a four-stranded anti-parallel β-sheet, their sequence similarities vary greatly; *i.e.*, from near identity to almost no similarity, indicating the high versatility and modularity of this lectin family [60]. Considering that multivalency forms an essential aspect of lectins, the β-propeller structure of these lectins could provide a good scaffold for engineering multivalent features.

*Aleuria aurantia* lectin (AAL) from orange peel mushroom is a homodimeric protein showing binding preference to oligosaccharides having terminal L-Fuc residues. In its crystal structure, each monomer is composed of a six-fold β-propeller structure with five different binding sites for L-Fuc. Considering the multivalent nature of AAL, it is anticipated that unwanted agglutination or precipitation may occur when targeting multivalent glycoproteins. In this context, Olausson *et al.* engineered two AAL variants by site-directed mutagenesis: one is a monomeric mutant, and the other is a mutant containing only one L-Fuc-binding site. Both exhibited reduced agglutination activity [61]. Tachylectin-2 is another five-bladed β-propeller lectin with five GlcNAc/GalNAc-binding sites, which is likely to be evolutionarily formed by gene duplication and subsequent fusion. Yadid *et al.* constructed a library of Tachylectin-2 by fusing the diversified sequence module in tandem, and a range of new lectins with sugar-binding activity were isolated from the library by a phage display method [62]. However, reproducing functional β-propellers by simple duplication and fusion of repeated units remains a challenging issue.

## 3. Key Points Associated with the Strategy of Lectin Engineering

### 3.1. Scaffold (Template)

Choice of a suitable lectin scaffold is the first key point for successful lectin engineering. Considering the practical application of engineered lectins for probes to particular glycans or disease therapy, they should be produced as easily as possible from a cost-effective viewpoint. Thus, the template lectin selected for engineering must be easily produced by common expression systems represented by *E. coli*. Yeast expression systems, such as the one using *Pichia pastoris* have proved to be useful for production of plant lectins, e.g., *Phaseolus vulgaris* agglutinin and snowdrop lectin, which are hard to be expressed in *E. coli* [63]. Of course, the expression system to be selected depends largely on the applications and purposes of the engineered lectin. With particular reference to when lectins are tailored as tools for glycan structural analysis, those that can be easily produced by *E. coli* are preferentially chosen as templates based on at least two reasons: (1) *E. coli* is able to produce non-glycosylated recombinant lectins that are more suitable for glycome profiling without influence from its own glycans. (2) *E.* *coli* is the most widely used expression system, and benefits from low cost and high productivity. Though L-type lectins are the best-studied, the production of these lectins in *E.* *coli* remains a challenge due to differences in post-translational features between plants and bacteria. C-type lectins are also difficult to produce in *E.* *coli*, possibly because of their conserved disulfide bonds. In contrast, a number of lectins from the galectin, R-type lectin, and β-propeller lectin families have been successfully produced in *E.* *coli*, suggesting they are suitable scaffolds for engineering. In our studies, we chose an R-type lectin (EW29Ch) and a fungus-derived galectin (ACG) as templates, and successfully tailored several lectins with practical utility [22,45,47,55]. Recently, more and more lectins have been discovered from microorganisms such as bacteria, algae and fungi, and most of them can be expressed well in *E. coli* [64,65]. These lectins will be a great source as scaffolds to expand diverse tools for glycome profiling.

In addition, the target activity to be engineered is also a key factor in template selection. Lectins often have a primary binding site for a certain monosaccharide, while they are often equipped with adjacent binding site(s) that define the fine glycan-binding specificity by recognition of the derivatives of the corresponding monosaccharide. Several studies have attempted to engineer lectins with altered monosaccharide specificity by modifying the primary binding site, but without success. In contrast, tuning the oligosaccharide specificity rather than change monosaccharide specificity is more effective by tailoring the adjacent binding site. As mentioned, R-type lectins are generally Gal/GalNAc-specific. X-ray crystallographic studies on extensive R-type lectins and their ligand β-galactosides have revealed that multiple hydroxyl groups of Gal (except for the C6 position) are involved in hydrogen bonding with proteins. Thus, R-type lectin can be used as an optimal template for engineering lectins for 6-*O*-modified Gal (e.g., Neu5Acα2-6Gal, 6-sulfo-galactose (6S-Gal)), with least destruction of the global structure. Based on this hypothesis, we have successfully engineered two lectin mutants with novel specificity, *i.e.*, SRC for α2-6-sialic acid and E20K for 6S-Gal from a Gal-specific R-type lectin EW29Ch [22]. Similarly, galectins are good templates for engineering 3-*O*-modified Gal (e.g., Neu5Acα2-3Gal, 3S-Gal) [45]. Thus, selection of a suitable template lectin is a key factor in the successful engineering of mutants with target activity.

### 3.2. Methods for Mutagenesis

Site-directed mutagenesis is the simplest and most commonly used method in lectin engineering, particularly in regards to lectins whose crystal structures have been elucidated with their glycan ligands. As described above, we recently conducted site-directed mutagenesis on a fungus galectin (ACG) by replacing each of the amino acid residues involved in the Gal-binding with Ala [45,47]. Among the five resulting mutants, two (N46A and E86A) acquired altered specificity. In particular, a single amino-acid substitution at position 46 (N46A) resulted in enhancement of the activity for GalNAcα1-3Galβ-containing glycans, while completely eliminating affinity to β-galactosides, which was accomplished by an unexpected mechanism involving the *cis*-*trans* isomerization regarding Pro45. In addition, E86A acquired a highly specific binding for 3'S-LacNAc in a “loss-of-function” rather than “gain-of-function” manner. These results suggest that structure-based site-directed mutagenesis is still an effective way to generate useful glycan probes.

Saturation mutagenesis is a practical extension of site-directed mutagenesis to optimize function by substituting a target amino acid with the remaining 19 amino acids. Imamura *et al.* carried out this strategy by targeting Asp86 in ACG, and tailored a mutant E86D with improved specificity for α2-3-sialic acid [43]. However, in that study, they failed to discover the specific binding ability of E86A to 3'S-LacNAc, which was revealed in our later study using glycoconjugate microarray [47], suggesting that adopting a high-throughput method for sugar-binding analysis is quite important for success in lectin engineering.

To further expand molecular diversity, a method called gene-scanning saturation mutagenesis has been developed: it is performed by subsequent substitution of each amino acid in a whole protein or a specific target region. Since the method has proved to be a powerful technique in protein engineering [66,67], its application in lectin engineering is worth pursuing.

Despite the success of site-directed mutagenesis, it is still difficult to predict the consequence of a certain mutation due to our limited understanding of the complex and cooperative network of weak interactions between glycans and lectins. To overcome this challenge, an approach termed directed evolution that mimicked the natural process of Darwinian evolution was developed, which involves generating molecular diversity by the introduction of random mutations within the target gene, followed by the selection of desired variants [68]. Error-prone PCR is one of the most commonly used approaches, which introduces random mutations during the procedure of PCR with a lowered fidelity of DNA polymerase; e.g., by adding manganese ions or biasing the deoxy-ribonucleotide triphosphate concentrations [69]. It is essentially simple, and without need for special techniques. We have recently used this method to generate a random mutagenesis library of the R-type lectin EW29Ch and succeeded in tailoring a useful mutant (E20K) as described above. However, error-prone PCR has the inherent limitation of codon and amplification biases during PCR, which will significantly reduce the genetic diversity achieved. To solve these problems, a method called Trinucleotide exchange (TriNEx) by random substitution of one contiguous trinucleotide sequence for another was recently established by Baldwin *et al.* [70]. Its application in the directed evolution of lectins is anticipated.

### 3.3. Methods for Screening

**Figure 3:**
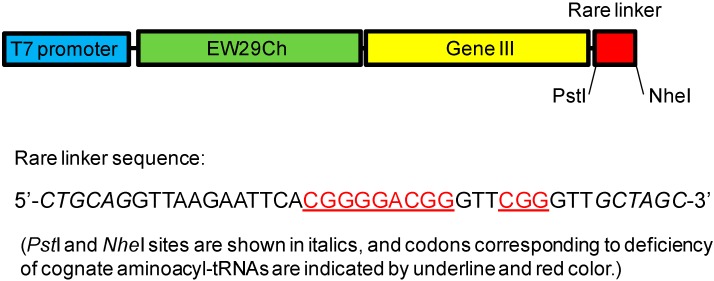
Diagram of the gene construct for ribosome display. *Pst*I and *Nhe*I sites are shown in italic. The sequence of the rare linker is shown below, where the codons corresponding to deficiency of cognate aminoacyl-tRNAs are indicated by underlining and red color.

With respect to the directed evolution of lectins, a high throughput screening method is also essential for the successful engineering and preparation of a high quality library. For this, there has been a variety of methods established in the past including phage display, bacterial display, yeast display, ribosome display and mRNA display, among others [71]. Ribosome display is an *in vitro* selection and evolution method for proteins from large libraries [72]. As it is carried out entirely *in vitro*, it has two major advantages compared with other selection methods: first, the diversity of the library is not limited by the transformation efficiency of bacterial cells, but only by the number of ribosomes and different mRNA molecules in the tube. Second, random mutations can be introduced easily after each selection round. These advantages permit the easy, directed evolution of binding proteins over several generations, which has been successfully used in engineering antibodies with high affinity (*K*_d_, ~10^−9^ M). However, since the interaction between lectin and glycan is generally much weaker than that between antibody and antigen, a longer time is required for the selection of lectins, during which key mRNA–ribosome–protein interactions may be disrupted. Thus, substantial stabilization of the complex is necessary for the application of this approach to lectin selection. Yabe *et al.* reported that incorporation of a rare codon sequence at the 3' region of the ribosome display construct sufficiently increased the stability of the mRNA-ribosome-protein complex based on the principle of ribosome stalling machinery (Figure 3) [55]. Recently, Hu *et al.* further refined this system by (1) using commercially available biotinylated carbohydrate polymers with a structure-defined, homogeneous glycan as a bait ligand instead of a glycoprotein for selection, which significantly reduced the background selection, (2) adding competent inhibitors during the selection process to enhance selection efficiency, and (3) adopting a glycoconjugate microarray system for rapid verification of the selected mutants. These modifications have greatly improved the efficiency of the ribosome display [22].

## 4. Future Perspectives

**Figure 4:**
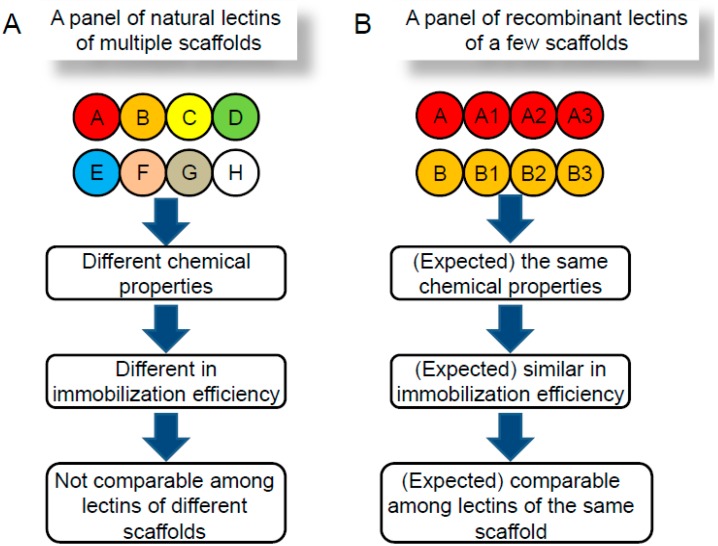
Schematic diagram of current lectin microarray and next generation lectin microarray. (**A**) Circles with different color and different upper-case letters represent different lectins. (**B**) Circles with the same color and same letter but different numbers represent lectins engineered from the same lectin template.

Glycomics and glycoproteomics have become of central interest in the post-genomic era. Development of effective methods for glycan analysis is therefore of great value. The sugar-binding ability of lectins has made them basic tools in glycomic studies. However, there are some inherent problems, and the limited repertoire of natural lectins does not fulfill the current requirements. Engineering lectins with improved properties is a promising way to achieve high quality and reproducibility. In this respect, lectins used in the current lectin microarray are different from one another in protein scaffold or protein family (Pfam). Thus, they may also have different specificities, structures and molecular weights (Figure 4A). In such a situation, it is difficult to immobilize all these lectins with the same efficiency (e.g., amount and orientation, *etc.*), even though they are spotted onto the chip at the same concentrations. This makes the current lectin microarray suitable only for comparing the binding profiles of different samples, but not among different lectins. On the other hand, lectins tailored from the same template (*i.e.*, protein family) are expected to show differences only in specificity, because they are equipped with almost the same chemical characteristics (e.g., structure and molecular weight). In Figure 4B, lectins derived from the same parent molecule are represented by circles with the same color and the same letter, but different numbers, wherein they are expected to be immobilized on the chip with the same efficiency in a compatible manner. The idea is conceptual at present, but with the next-generation lectin microarray systems, not only profile comparison among different samples but also comparison of the lectins tailored from the same template will be possible. As described, we successfully engineered a Gal-specific lectin (EW29Ch) to a mutant (E20K) capable of binding 6S-Gal. While the mutant retained Gal affinity, specific detection of 6S-Gal on cells can be achieved by the combinational use of EW29Ch and E20K, since they have the same protein scaffold and thus are compatible. If we see increased binding of E20K to cells compared with EW29Ch, it is very likely that these cells contain 6S-Gal. We consistently observed a stronger binding of E20K than EW29Ch to cells in which 6S-Gal was over-expressed (Figure 2). This study indicates that comparison of the binding profile of different lectins with the same scaffold could provide important information for glycan structural characterization.

Recent studies have proved the power of directed evolution in tailoring useful probes. With the advancement of high-throughput technologies including glycan microarray, it can be expected that many more lectins of practical use will be created in the near future.

## Abbreviations

Gal: galactose
GalNAc: N-acetylgalactosamine
Glc: glucose
GlcNAc: *N*-acetylglucosamine
Man: mannose
Fuc: fucose
Neu5Ac: *N*-Acetyl neuraminic acid

## References

[1] Hedlund M., Ng E., Varki A., Varki N.M. (2008). alpha 2–6-Linked sialic acids on N-glycans modulate carcinoma differentiation *in vivo*. Cancer Res..

[2] Tateno H., Toyota M., Saito S., Onuma Y., Ito Y., Hiemori K., Fukumura M., Matsushima A., Nakanishi M., Ohnuma K. (2011). Glycome diagnosis of human induced pluripotent stem cells using lectin microarray. J. Biol. Chem..

[3] Hirabayashi J. (2004). Lectin-based structural glycomics: Glycoproteomics and glycan profiling. Glycoconj. J..

[4] Hirabayashi J., Kuno A., Tateno H. (2011). Lectin-based structural glycomics: A practical approach to complex glycans. Electrophoresis.

[5] Krishnamoorthy L., Mahal L.K. (2009). Glycomic analysis: An array of technologies. ACS Chem. Biol..

[6] Zaia J. (2008). Mass spectrometry and the emerging field of glycomics. Chem. Biol..

[7] Hasehira K., Tateno H., Onuma Y., Ito Y., Asashima M., Hirabayashi J. (2012). Structural and quantitative evidence for dynamic glycome shift on production of induced pluripotent stem cells. Mol. Cell. Proteomics.

[8] Campbell M.P., Royle L., Radcliffe C.M., Dwek R.A., Rudd P.M. (2008). GlycoBase and autoGU: Tools for HPLC-based glycan analysis. Bioinformatics.

[9] Kuno A., Uchiyama N., Koseki-Kuno S., Ebe Y., Takashima S., Yamada M., Hirabayashi J. (2005). Evanescent-field fluorescence-assisted lectin microarray: A new strategy for glycan profiling. Nat. Methods.

[10] Pilobello K.T., Slawek D.E., Mahal L.K. (2007). A ratiometric lectin microarray approach to analysis of the dynamic mammalian glycome. Proc. Natl. Acad. Sci. USA.

[11] Tateno H., Nakamura-Tsuruta S., Hirabayashi J. (2007). Frontal affinity chromatography: Sugar-protein interactions. Nat. Protoc..

[12] Stevens J., Blixt O., Glaser L., Taubenberger J.K., Palese P., Paulson J.C., Wilson I.A. (2006). Glycan microarray analysis of the hemagglutinins from modern and pandemic influenza viruses reveals different receptor specificities. J. Mol. Biol..

[13] Hu D., Kamiya Y., Totani K., Kamiya D., Kawasaki N., Yamaguchi D., Matsuo I., Matsumoto N., Ito Y., Kato K. (2009). Sugar-binding activity of the MRH domain in the ER alpha-glucosidase II beta subunit is important for efficient glucose trimming. Glycobiology.

[14] Kawasaki N., Ichikawa Y., Matsuo I., Totani K., Matsumoto N., Ito Y., Yamamoto K. (2008). The sugar-binding ability of ERGIC-53 is enhanced by its interaction with MCFD2. Blood.

[15] Kawasaki N., Matsuo I., Totani K., Nawa D., Suzuki N., Yamaguchi D., Matsumoto N., Ito Y., Yamamoto K. (2007). Detection of weak sugar binding activity of VIP36 using VIP36-streptavidin complex and membrane-based sugar chains. J. Biochem..

[16] Mikami K., Yamaguchi D., Tateno H., Hu D., Qin S.Y., Kawasaki N., Yamada M., Matsumoto N., Hirabayashi J., Ito Y. (2010). The sugar-binding ability of human OS-9 and its involvement in ER-associated degradation. Glycobiology.

[17] Yamaguchi D., Kawasaki N., Matsuo I., Totani K., Tozawa H., Matsumoto N., Ito Y., Yamamoto K. (2007). VIPL has sugar-binding activity specific for high-mannose-type N-glycans, and glucosylation of the alpha1,2 mannotriosyl branch blocks its binding. Glycobiology.

[18] Onuma Y., Tateno H., Hirabayashi J., Ito Y., Asashima M. (2013). rBC2LCN, a new probe for live cell imaging of human pluripotent stem cells. Biochem. Biophys. Res. Commun..

[19] Tateno H., Onuma Y., Ito Y. (2014). Live-cell imaging of human pluripotent stem cells by a novel lectin probe rBC2LCN. Methods Mol. Biol..

[20] Kuno A., Ikehara Y., Tanaka Y., Angata T., Unno S., Sogabe M., Ozaki H., Ito K., Hirabayashi J., Mizokami M. (2011). Multilectin assay for detecting fibrosis-specific glyco-alteration by means of lectin microarray. Clin. Chem..

[21] Hsu K.L., Gildersleeve J.C., Mahal L.K. (2008). A simple strategy for the creation of a recombinant lectin microarray. Mol. Biosyst..

[22] Hu D., Tateno H., Kuno A., Yabe R., Hirabayashi J. (2012). Directed evolution of lectins with sugar-binding specificity for 6-sulfo-galactose. J. Biol. Chem..

[23] Sharon N., Lis H. (1990). Legume lectins—A large family of homologous proteins. FASEB J..

[24] Arango R., Rodriguez-Arango E., Adar R., Belenky D., Loontiens F.G., Rozenblatt S., Sharon N. (1993). Modification by site-directed mutagenesis of the specificity of Erythrina corallodendron lectin for galactose derivatives with bulky substituents at C-2. FEBS Lett..

[25] Jordan E.T., Goldstein I.J. (1995). Site-directed mutagenesis studies on the lima bean lectin. Altered carbohydrate-binding specificities result from single amino acid substitutions. Eur. J. Biochem..

[26] Thamotharan S., Karthikeyan T., Kulkarni K.A., Shetty K.N., Surolia A., Vijayan M., Suguna K. (2011). Modification of the sugar specificity of a plant lectin: Structural studies on a point mutant of Erythrina corallodendron lectin. Acta Crystallogr. Sect. D Biol. Crystallogr..

[27] Adhikari P., Bachhawat-Sikder K., Thomas C.J., Ravishankar R., Jeyaprakash A.A., Sharma V., Vijayan M., Surolia A. (2001). Mutational analysis at Asn-41 in peanut agglutinin—A residue critical for the binding of the tumor-associated Thomsen-Friedenreich antigen. J. Biol. Chem..

[28] Yamamoto K., Konami Y., Osawa T., Irimura T. (1992). Alteration of the carbohydrate-binding specificity of the Bauhinia purpurea lectin through the preparation of a chimeric lectin. J. Biochem..

[29] Yamamoto K., Konami Y., Osawa T. (2000). A chimeric lectin formed from Bauhinia purpurea lectin and Lens culinaris lectin recognizes a unique carbohydrate structure. J. Biochem..

[30] Nishiguchi M., Yoshida K., Sumizono T., Tazaki K. (1997). Studies by site-directed mutagenesis of the carbohydrate-binding properties of a bark lectin from Robinia pseudoacacia. FEBS Lett..

[31] Yamamoto K., Maruyama I.N., Osawa T. (2000). Cyborg lectins: Novel leguminous lectins with unique specificities. J. Biochem..

[32] Maenuma K., Yim M., Komatsu K., Hoshino M., Tachiki-Fujioka A., Takahashi K., Hiki Y., Bovin N., Irimura T. (2009). A library of mutated Maackia amurensis hemagglutinin distinguishes putative glycoforms of immunoglobulin A1 from IgA nephropathy patients. J. Proteome Res..

[33] Maenuma K., Yim M., Komatsu K., Hoshino M., Takahashi Y., Bovin N., Irimura T. (2008). Use of a library of mutated Maackia amurensis hemagglutinin for profiling the cell lineage and differentiation. Proteomics.

[34] Streicher H., Sharon N. (2003). Recombinant plant lectins and their mutants. Methods Enzymol..

[35] Barondes S.H., Cooper D.N., Gitt M.A., Leffler H. (1994). Galectins. Structure and function of a large family of animal lectins. J. Biol. Chem..

[36] Leffler H., Carlsson S., Hedlund M., Qian Y., Poirier F. (2004). Introduction to galectins. Glycoconj. J..

[37] Klyosov A.A., Traber P.G. (2012). Galectins in Disease and Potential Therapeutic Approaches. ACS Symp. Ser..

[38] Nishi N., Itoh A., Fujiyama A., Yoshida N., Araya S., Hirashima M., Shoji H., Nakamura T. (2005). Development of highly stable galectins: Truncation of the linker peptide confers protease-resistance on tandem-repeat type galectins. FEBS Lett..

[39] Itoh A., Fukata Y., Miyanaka H., Nonaka Y., Ogawa T., Nakamura T., Nishi N. (2013). Optimization of the inter-domain structure of galectin-9 for recombinant production. Glycobiology.

[40] Wang H., He L., Lensch M., Gabius H.J., Fee C.J., Middelberg A.P. (2008). Single-site Cys-substituting mutation of human lectin galectin-2: Modulating solubility in recombinant production, reducing long-term aggregation, and enabling site-specific monoPEGylation. Biomacromolecules.

[41] Kopitz J., Fik Z., Andre S., Smetana K., Gabius H.J. (2013). Single-site mutational engineering and following monoPEGylation of the human lectin galectin-2: Effects on ligand binding, functional aspects, and clearance from serum. Mol. Pharm..

[42] Ban M., Yoon H.J., Demirkan E., Utsumi S., Mikami B., Yagi F. (2005). Structural basis of a fungal galectin from Agrocybe cylindracea for recognizing sialoconjugate. J. Mol. Biol..

[43] Imamura K., Takeuchi H., Yabe R., Tateno H., Hirabayashi J. (2011). Engineering of the glycan-binding specificity of Agrocybe cylindracea galectin towards alpha(2,3)-linked sialic acid by saturation mutagenesis. J. Biochem..

[44] Yagi F., Hiroyama H., Kodama S. (2001). Agrocybe cylindracea lectin is a member of the galectin family. Glycoconj. J..

[45] Hu D., Tateno H., Sato T., Narimatsu H., Hirabayashi J. (2013). Tailoring GalNAc alpha 1–3Gal beta-specific lectins from a multi-specific fungal galectin: Dramatic change of carbohydrate specificity by a single amino-acid substitution. Biochem. J..

[46] Kuwabara N., Hu D., Tateno H., Makyio H., Hirabayashi J., Kato R. (2013). Conformational change of a unique sequence in a fungal galectin from Agrocybe cylindracea controls glycan ligand-binding specificity. FEBS Lett..

[47] Hu D., Huang H., Tateno H., Nakakita S., Sato T., Narimatsu H., Yao X., Hirabayashi J. (2015). Engineering of a 3'-sulpho-Galbeta1–4GlcNAc-specific probe by a single amino acid substitution of a fungal galectin. J. Biochem..

[48] Ryckaert S., Callewaert N., Jacobs P.P., Dewaele S., Dewerte I., Contreras R. (2008). Fishing for lectins from diverse sequence libraries by yeast surface display—An exploratory study. Glycobiology.

[49] Hemmi H., Kuno A., Ito S., Suzuki R., Hasegawa T., Hirabayashi J. (2009). NMR studies on the interaction of sugars with the C-terminal domain of an R-type lectin from the earthworm Lumbricus terrestris. FEBS J..

[50] Shibuya N., Goldstein I.J., Broekaert W.F., Nsimba-Lubaki M., Peeters B., Peumans W.J. (1987). The elderberry (*Sambucus nigra* L.) bark lectin recognizes the Neu5Ac(alpha 2–6)Gal/GalNAc sequence. J. Biol. Chem..

[51] Kaku H., Tanaka Y., Tazaki K., Minami E., Mizuno H., Shibuya N. (1996). Sialylated oligosaccharide-specific plant lectin from Japanese elderberry (*Sambucus sieboldiana*) bark tissue has a homologous structure to type II ribosome-inactivating proteins, ricin and abrin. cDNA cloning and molecular modeling study. J. Biol. Chem..

[52] Tateno H., Mori A., Uchiyama N., Yabe R., Iwaki J., Shikanai T., Angata T., Narimatsu H., Hirabayashi J. (2008). Glycoconjugate microarray based on an evanescent-field fluorescence-assisted detection principle for investigation of glycan-binding proteins. Glycobiology.

[53] Fiete D.J., Beranek M.C., Baenziger J.U. (1998). A cysteine-rich domain of the “mannose” receptor mediates GalNAc-4-SO4 binding. Proc. Natl. Acad. Sci. USA.

[54] Hirabayashi J., Dutta S.K., Kasai K. (1998). Novel galactose-binding proteins in Annelida. Characterization of 29-kDa tandem repeat-type lectins from the earthworm Lumbricus terrestris. J. Biol. Chem..

[55] Yabe R., Suzuki R., Kuno A., Fujimoto Z., Jigami Y., Hirabayashi J. (2007). Tailoring a novel sialic acid-binding lectin from a ricin-B chain-like galactose-binding protein by natural evolution-mimicry. J. Biochem..

[56] Yabe R., Itakura Y., Nakamura-Tsuruta S., Iwaki J., Kuno A., Hirabayashi J. (2009). Engineering a versatile tandem repeat-type alpha 2-6sialic acid-binding lectin. Biochem. Biophys. Res. Commun..

[57] Drickamer K. (1992). Engineering galactose-binding activity into a C-type mannose-binding protein. Nature.

[58] Michelow I.C., Dong M., Mungall B.A., Yantosca L.M., Lear C., Ji X., Karpel M., Rootes C.L., Brudner M., Houen G. (2010). A novel L-ficolin/mannose-binding lectin chimeric molecule with enhanced activity against Ebola virus. J. Biol. Chem..

[59] Chang W.C., Hartshorn K.L., White M.R., Moyo P., Michelow I.C., Koziel H., Kinane B.T., Schmidt E.V., Fujita T., Takahashi K. (2011). Recombinant chimeric lectins consisting of mannose-binding lectin and L-ficolin are potent inhibitors of influenza A virus compared with mannose-binding lectin. Biochem. Pharmacol..

[60] Chaudhuri I., Soding J., Lupas A.N. (2008). Evolution of the beta-propeller fold. Proteins.

[61] Olausson J., Astrom E., Jonsson B.H., Tibell L.A.E., Pahlsson P. (2011). Production and characterization of a monomeric form and a single-site form of Aleuria aurantia lectin. Glycobiology.

[62] Yadid I., Tawfik D.S. (2011). Functional beta-propeller lectins by tandem duplications of repetitive units. Protein Eng. Des. Sel..

[63] Raemaekers R.J., de Muro L., Gatehouse J.A., Fordham-Skelton A.P. (1999). Functional phytohemagglutinin (PHA) and Galanthus nivalis agglutinin (GNA) expressed in Pichia pastoris correct N-terminal processing and secretion of heterologous proteins expressed using the PHA-E signal peptide. Eur. J. Biochem..

[64] Propheter D.C., Hsu K.L., Mahal L.K. (2011). Recombinant lectin microarrays for glycomic analysis. Methods Mol. Biol..

[65] Topin J., Arnaud J., Sarkar A., Audfray A., Gillon E., Perez S., Jamet H., Varrot A., Imberty A., Thomas A. (2013). Deciphering the glycan preference of bacterial lectins by glycan array and molecular docking with validation by microcalorimetry and crystallography. PLoS ONE.

[66] You C., Percival Zhang Y.H. (2012). Easy preparation of a large-size random gene mutagenesis library in *Escherichia coli*. Anal. Biochem..

[67] Krauss U., Jaeger K.E., Eggert T. (2010). Rapid sequence scanning mutagenesis using in silico oligo design and the Megaprimer PCR of whole plasmid method (MegaWHOP). Methods Mol. Biol..

[68] Nannemann D.P., Birmingham W.R., Scism R.A., Bachmann B.O. (2011). Assessing directed evolution methods for the generation of biosynthetic enzymes with potential in drug biosynthesis. Future Med. Chem..

[69] McCullum E.O., Williams B.A., Zhang J., Chaput J.C. (2010). Random mutagenesis by error-prone PCR. Methods Mol. Biol..

[70] Baldwin A.J., Busse K., Simm A.M., Jones D.D. (2008). Expanded molecular diversity generation during directed evolution by trinucleotide exchange (TriNEx). Nucleic Acids Res..

[71] Lin H., Cornish V.W. (2002). Screening and selection methods for large-scale analysis of protein function. Angew. Chem. Int. Ed..

[72] He M., Khan F. (2005). Ribosome display: Next-generation display technologies for production of antibodies *in vitro*. Expert Rev. Proteomics.

